# Prenatal nursing intervention studies published in Korean nursing journals: a scoping review

**DOI:** 10.4069/kjwhn.2020.06.12

**Published:** 2020-06-22

**Authors:** Seo Yun Kim, Hae Won Kim

**Affiliations:** 1 College of Nursing, Seoul National University, Seoul, Korea; 2 College of Nursing, Research Institute of Nursing Science, Seoul National University, Seoul, Korea

**Keywords:** Nursing care, Nursing research, Pregnancy, Prenatal care, Review literature

## Abstract

The purpose of this study was to describe prenatal nursing intervention studies on pregnant women and their families published in Korean nursing journals to identify research trends and to analyze the characteristics of intervention studies. This scoping review was conducted using Arksey and O’Malley’s framework. We identified a research question and searched six domestic electronic databases for relevant articles. Forty-five references that met the inclusion and exclusion criteria were finally selected. We extracted the data using an analytic framework, and then collated and summarized the characteristics of the intervention studies. The most frequently used research designs were non-randomized controlled trials (91.1%), and only a few studies applied a specific theoretical framework (24.4%). The participants were mainly pregnant women only (64.4%) during the third trimester (35.6%) of pregnancy. Prenatal education was the most common type of intervention (48.9%), followed by complementary therapy (37.8%) and psychosocial support programs (13.3%). The most commonly used outcome variables were drawn from the psychological domain (44.5%), although distinct types of outcome variables—especially from the psychological and physical domains—were used to measure the effectiveness of different types of prenatal interventions. This review suggests that further prenatal nursing intervention studies in Korea should expand the study participants to include pregnant women’s family members, high-risk and vulnerable groups, and women throughout entire pregnancy. Furthermore, it is necessary to develop integrative prenatal nursing interventions that promote family support and participation by facilitating partnerships among women, families, and nurses before, during, and after pregnancy.

## Introduction

Prenatal care (PNC) is vital for healthy pregnancy outcomes [1]. Effective PNC has been reported to reduce maternal mortality and negative birth outcomes [2,3], and the World Health Organization (WHO) emphasizes timely, evidence-based, high-quality PNC [3].

Although healthcare providers and policy-makers have sought to increase engagement in PNC worldwide, participation in PNC remains poor [4,5]. Nurses play a unique role in conducting PNC research and developing interventions for pregnant women and their families [6]. To date, integrated research related to prenatal or postpartum care in Korea has included a study of the effect of intervention programs on improving maternal adaptation among postpartum women, an integrative review of the evidence regarding home care service interventions for mothers and children in vulnerable groups, a review of current quantitative research on maternal adaptation among married immigrant women, and an analysis of research trends on pregnancy and childbirth among married immigrant women [7-10]. However, no study has comprehensively synthesized the results of prenatal nursing intervention (PNI) studies. In light of current social and cultural issues such as low birth rate, infertility, high-risk pregnancies, and increasing number of marriage immigrants, a comprehensive understanding of PNI studies that have conducted to date will be helpful for planning tailored PNIs based on individuals’ specific nursing needs. Therefore, this scoping study aimed to identify research trends, to synthesize meaningful results from previous PNI studies on pregnant women and their families in Korea, and to suggest directions for the future development of PNIs.

### Objectives

The purpose of this study was to identify trends in PNI research conducted among pregnant women and their families in Korea, and to analyze the characteristics of the studies to suggest future directions in PNI research. The specific goals of this study were 1) to assess the characteristics of the selected studies, 2) to analyze the characteristics of the interventions and the outcome variables, and 3) to identify the effects of interventions.

## Methods

Ethics statement: This study is a literature review of previously published studies and was therefore exempt from approval of Institutional Review Board and informed consent from the subjects.

### Study design

This study was conducted in five stages according to the scoping review methodological framework suggested by Arksey and O’Malley [11] in 2005: 1) identifying the research question, 2) searching for relevant studies, 3) selecting studies for inclusion, 4) charting the data, and 5) collating, summarizing, and reporting the results.

### Search and selection of studies

#### Identifying the research question

The research question was “What are the characteristics and effects of intervention studies related to prenatal care published in Korean nursing journals?”

#### Searching for relevant studies

##### Search strategy

The key question for the literature search was specified based on the population, intervention, comparisons, outcomes (PICO) framework, as follows: 1) pregnant women and families (P), 2) nursing interventions related to PNC (I), 3) a comparator group was not provided with PNI (C), and 4) one or more outcome variables showed statistically significant differences (O). No restrictions were placed on the publication year of the studies. Only studies conducted in Korea and published in Korean nursing journals in Korean or English with accessible full texts were included in the analysis. Non-experimental studies were excluded.

##### Data collection

Data were collected in two stages. The primary search was conducted between April 2 and April 15, 2019, using six Korean databases: Research Information Sharing Services (RISS), Korean studies Information Service System (KISS), National Digital Science Library (NDSL), DBpia, KMbase, and KoreaMed. The preliminary search was conducted based on the key question (PICO). After reviewing the retrieved abstracts, a comprehensive search strategy was established. The final selected search terms (“pregnant,” “pregnant women,” “prenatal,” “antenatal,” “prepared childbirth,” “before childbirth,” “preconception,” “pre-pregnancy,” “before pregnancy,” “healthy pregnancy”) and (“nursing,” “care,” “health promotion,” “health management,” “education,” “counseling,” “program”) were combined and searched. The results were filtered by title, abstract, and keyword, and in KISS, DBpia, and RISS, the results were limited to the pharmaceutical and nursing fields. The search period included all studies published before April 2, 2019. Considering the time required for analysis and manuscript writing, it was decided by the researchers that an additional search was necessary. On January 8, 2020, a second search was conducted of 67 domestic nursing journals [12] in KMbase. All papers published from April to December 2019 were obtained and further reviewed in the same manner.

#### Selecting studies to include

**Figure. 1.**
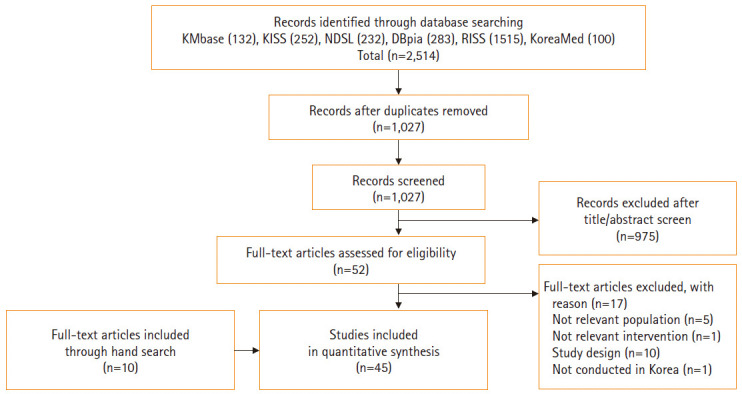
Flow chart of study selection.

Duplicate exclusion and initial screening based on the title and abstract reduced the list of obtained papers from 2,514 to 1,027. After excluding articles that did not meet the selection criteria, 52 full texts were reviewed. Thirty-five studies were selected, eliminating five articles that did not deal with the relevant population, one that did not deal with PNI, 10 that were not experimental studies or were program development studies without research results, and one that was not conducted in Korea. Ten other articles were added from the references in the selected literature. No papers were added in the second search, yielding a final 45 papers to be reviewed (Figure 1, Supplement 1). The selection and elimination process was independently conducted by two researchers, and disagreements were resolved via discussion.

##### Charting the data

An analytical framework was developed to analyze and extract the characteristics of the analyzed studies based on criteria used in previous research (Supplement 2), including general characteristics (author, publication year, research design, type of research, use of theoretical framework), characteristics of the participants (intervention scope, primipara or multipara, pregnancy period, high-risk pregnancy factors, sample size) and the intervention (name of intervention, contents, setting, unit, methods, experimental period, number of sessions, time per session, follow-up period), and outcome variables.

To facilitate analyses of research trends over time, the studies were divided into 10-year units from 1980 to 2019. The study design was classified using the Study Design Algorithm for Medical Literature of Intervention (DAMI) tool [13].

Interventions were classified as occurring in the first trimester (≤14 weeks), second trimester (15 to 26 weeks), third trimester (27 to 42 weeks), or during labor and birth. High-risk pregnancy factors were categorized as obstetric, medical, physical, and current pregnancy risk factors based on the Korean Society of Obstetrics and Gynecology classification [14]. Information on specific groups (cultural and racial groups or vulnerable groups) was also added.

PNIs were classified as health education, psychosocial support, and complementary therapy [15]. The outcome variables were divided into five domains by adding the physiological domain to the psychological, interpersonal, perceptual, and behavioral domains [9].

Collected data were analyzed by frequency and percentage using Excel 2016 (Microsoft, Redmond, WA, USA).

## Results

### General attributes of selected literature

#### General characteristics of the selected studies

Of the 45 studies analyzed in this study, five were published between 1980 and 1989, three between 1990 and 1999, fifteen between 2000 and 2009, and twenty-two between 2010 and 2019. The most common study design was non-randomized controlled trials (n=41, 91.1%). Only 12 studies (26.1%) applied a theoretical framework (Table 1).

#### Participant descriptions

Twenty-nine studies (64.4%) included pregnant women only, nine (20.0%) included pregnant women and spouses, and three (6.7%) included pregnant women and infants. Research with an expanded scope, including infants and families, has been published since 2000. Twenty-two articles (48.9%) were conducted among primiparae, while 23 (51.1%) included primiparae and multiparae. Since 2000, the participants have expanded to include both primiparae and multiparae. Most interventions were performed during pregnancy (n=38, 84.4%), five were during labor and birth (11.1%), and two were during both pregnancy and labor and birth (4.4%). The third trimester was the most common timing during pregnancy (n=23, 51.1%). Since 2000, interventional studies have been conducted at other times, including the first trimester.

The high-risk pregnancy factors of the participants included preterm labor (n=4, 8.7%), gestational diabetes (n=3, 6.5%), and cesarean section (n=2, 4.4%). One study dealt with immigrant women (2.2%). Eleven studies (78.6%) were conducted among high-risk pregnant women and their families between 2010 and 2019.

Twenty-two studies (48.9%) had fewer than 30 participants in the experimental and control groups (Table 1).

### Characteristics of PNIs and outcome variables

#### Characteristics of PNIs

Interventions were performed in hospital settings in 39 studies (86.7%). Twenty-one studies (46.7%) used individualized interventions, whereas 18 (39.1%) used group interventions, and five (10.9%) involved a combination. Instruction was the most frequent intervention method (n=34, 37.8%). The program duration ranged from 1 day to 12 weeks; interventions were provided for 3 to 6 weeks in 18 studies (40.0%) and 1 day in 11 studies (24.4%). Twenty studies (44.4%) had 1 to 4 sessions. The most common duration was ≤60 minutes (n=24, 53.3%). Nineteen studies (42.2%) conducted follow-up immediately after the intervention (Table 2).

#### Intervention type

Prenatal health education was the most common intervention type (n=22, 48.9%), followed by complementary therapy (n=17, 37.8%) and psychosocial support (n=6, 13.3%) (Table 2). Prenatal education was classified into education on pregnancy-related health care and childbirth (n=12, 13.2%), high-risk pregnancy health care (n=4, 8.9%), newborn care and the parental role (n=3, 6.5%), breastfeeding and care (n=2, 4.5%); and health-related behavior (n=1, 2.2%). Complementary therapy was divided into relaxation/abdominal breathing, music therapy and yoga/qigong training (each n=4, 8.9%), therapeutic touch/massage and acupressure (each n=2, 4.5%), and aromatherapy (n=1, 2.2%). Psychosocial support was classified as family participation in the delivery and reinforcement of spousal support (each n=3, 6.5%) (Table 2).

#### Outcome variables

One to seven outcome variables were used to measure the effectiveness of PNIs, with a total of 57 variables (44.5%) belonging to the psychological, 42 (32.8%) in physiological domains. Anxiety was the most frequently measured variable (n=21) although studies have diversified to explore depression, stress, confidence, and self-efficacy since 2000. In the physiological domain, labor pain was the most commonly evaluated variable (n=9), although studies expanded to include physical signs, symptoms, and biochemical parameters since 2000. Perceptions of childbirth experience (n=5), maternal-fetal attachment (n=3), and breastfeeding-related variables (n=3) were the most frequently measured variables in the perceptual, interpersonal, and behavioral domains, respectively (Table 3).

### Effects of interventions

#### Prenatal health education

Of the 22 studies dealing with prenatal health education, pregnancy-related health care and childbirth education was included in 12 studies, of which three focused on the normal course of pregnancy and childbirth, one on coaching in childbirth, one on experience-focused prenatal education, six on the Lamaze method, and one on sophrologic prenatal education. The main findings were significantly reduced anxiety (5 of 7), increased childbirth confidence and self-efficacy (3 of 3), and reduced labor pain (3 of 4).

High-risk pregnancy health care education was addressed in four studies, of which three focused on coaching-based health management programs for women with gestational diabetes and one focused on web-based prenatal education for advanced maternal age. The main findings were meaningful enhancements of self-efficacy (2 of 2), self-management or health care behavior (3 of 3), and reductions in depression (2 of 2) and anxiety (1 of 1).

Three studies involved interventions dealing with newborn care and parental role education, including one study focusing on newborn care for married immigrant women, another on a program to promote mother-fetus interaction, and the other was parental role education for primiparae. The main results were positive changes in childrearing self-efficacy, maternal role self-confidence, and mother-infant interactions (1 of 1 for each).

Breastfeeding and care education was implemented in two studies, with a significant increase in breastfeeding rate and practices (2 of 2).

Health-related behavior education was applied in one study, in which an oral health and walking exercise program for healthy pregnant women reduced depression and stress, and led to significantly improved quality of life and oral health behavior.

#### Complementary therapy

Of the 17 studies dealing with complementary therapy, four investigated relaxation and abdominal breathing. Of these, three studies were conducted among pregnant women with preterm labor, and one among primiparae during normal labor and birth. Significant positive effects were found on reducing stress (2 of 2), anxiety (4 of 4), and tocolytic dosage (1 of 1), as well as on stable vital signs (2 of 2).

Music therapy was used in four studies, of which two were conducted among healthy pregnancy women during a non-stress test or transvaginal ultrasound, one among pregnant women with preterm labor, and one among women undergoing a cesarean section. Significant improvements in anxiety (3 of 3) and preterm labor stress (1 of 1) were reported.

Yoga and qigong training was included in four studies, with significant effects on reducing labor pain (2 of 3) and postpartum discomfort (1 of 1); promoting a normal body mass index (1 of 1) and birth weight (1 of 1); reducing anxiety (2 of 3), pregnancy stress (1 of 1), and depression (1 of 1).

Two studies using therapeutic touch and massage showed positive effects on anxiety, spousal support, perceptions of the childbirth experience, paternal role confidence, and couple attachment (1 of 1 for each). Two studies using acupressure showed significant differences in blood cortisol levels and in nausea and vomiting (1 of 1 for each). One study applied aromatherapy to high-risk pregnant women and found significant effects on reducing antenatal stress.

#### Psychosocial support

Six studies investigated psychosocial support, of which three studies provided spousal support reinforcement during the normal process of labor and delivery, while three studies applied programs promoting family participation in delivery. The main results were positive perceptions of the childbirth experience (2 of 3), self-efficacy regarding childbirth (1 of 1), spousal support (2 of 2), spousal participation (1 of 1) and reduced antenatal stress (1 of 1).

## Discussion

This study identified research trends and analyzed the characteristics of PNIs among pregnant women and their families in Korea.

Most studies were non-random experimental studies, and only a handful used specific theoretical frameworks; however, it is encouraging that the number of randomized experimental studies and studies using theoretical frameworks has increased since 2010.

Although the study participants were mainly limited to pregnant women, studies gradually expanded to include infants, spouses, and entire families. This is a favorable trend in light of research demonstrating that support from spouses and family members during pregnancy and childbirth has a significant effect on pregnant women’s birth experiences [16,17]. Primiparae were the main participants because of their high levels of fear and anxiety as they prepare for childbirth and labor pain [18]. Since 2000, the number of intervention studies involving both primiparae and multiparae has increased, presumably due to the selection of participants according to nursing needs. Interventions were most frequently performed during the second and third trimesters; however, PNC in the early stages of pregnancy has been proposed as a way to predict unfavorable birth outcomes [19]. Therefore, nursing interventions at various time points in pregnancy should be studied, including the first trimester.

Studies of high-risk pregnancies have become increasingly common due to increasing rates of late pregnancy and childbirth [20]. Furthermore, as the number of marriage immigrants is steadily increasing [20], more comprehensive PNIs should be provided for them.

Interventions were most commonly provided at hospitals. Reasons for not participating in prenatal education include limitations of time and place [4,5]. Due to the insufficient availability of prenatal education at public health centers, prenatal education sessions are now frequently held by department store cultural centers, private companies, or online [21]. Therefore, interventions should consider the accessibility and convenience of PNC. Group interventions were frequently performed in older studies, but individual and mixed interventions became more frequent. A group PNC model known as ‘CenteringPregnancy’ has been developed in the United States and applied with the intention of improving perinatal outcomes [22]. However, in a recent Cochrane review, the effects of group and individual pregnancy management were compared through a randomized controlled study, and no significant difference was found in major pregnancy outcomes [23]. According to the WHO guideline for PNC, individual and group interventions should be selected according to individual preferences [3]. Therefore, the needs and cost-effectiveness of individual and group interventions should be considered in future intervention studies. Most interventions involved short sessions, with follow-up conducted immediately post-intervention. Longitudinal studies should investigate longer-term intervention strategies to promote ongoing healthy behaviors even after childbirth.

Many studies used psychological outcome variables, such as anxiety, and physiological outcome variables, such as labor pain. In addition, there was a tendency to use objective values, such as physiological measurements and clinical signs and symptoms. Objective measurements support evidence-based nursing and increase the validity of the research [24].

The most widely used type of intervention was prenatal health education, which is a key element of PNIs. In most studies, prenatal education had a significant effect on reducing anxiety, improving self-efficacy and self-confidence, and alleviating labor pain. These results are similar to findings of international research that systematic prenatal education was effective in improving knowledge and promoting self-efficacy and psychosocial well-being [25]. However, there was a lack of education related to health-promoting behaviors. Thus, PNIs should be developed that focus on healthy behaviors before, during, and after pregnancy, with regular follow-up to verify their long-term effects.

Nursing studies with complementary interventions gradually became more frequent. It has been reported that 51% to 68% of women in advanced Western countries received alternative therapies during pregnancy [26-28]. In this study, complementary therapy reduced anxiety, stress, and physical discomfort or labor pain; therefore, future research should explore standardization of complementary therapy for safe clinical practice.

Psychosocial support interventions enhanced spousal support and participation and improved perceptions of childbirth experience and self-efficacy in delivery. Previous studies have suggested that continuous support during labor, particularly spousal support, is the strongest predictors of a mother’s positive childbirth experience [16,29].

The significance of this study is that it identified research trends and analyzed the characteristics of intervention studies that applied PNIs to pregnant women and families in Korea. Thus, it provides baseline evidence and suggests directions for effective PNI development in the future. However, it may have been affected by publication bias and we could not assess the methodological quality of the included studies.

This study analyzed research trends of PNIs conducted among pregnant women and their families. The following specific directions should be considered for future research. First, researchers should increase the level of evidence by conducting randomized controlled trials. Second, PNI research should be expanded beyond women in the second and third trimesters of pregnancy, and should also account for various high-risk factors. Third, integrated interventions should be developed with the goal of establishing effective partnerships among women, families, and nurses and promoting family support and participation throughout the pregnancy. Fourth, standardized processes should be established for presenting evidence and implementing effective PNIs. Finally, highly accessible interventions should be developed using modern media.

## Figures and Tables

**Table 1.** General characteristics of the selected studies (N=45)

Variable	Categories	Total	1980–1989	1990–1999	2000–2009	2010–2019
			n=5	n=3	n=15	n=22
Research design	Randomized controlled trial	3 (6.7)	0 (0.0)	0 (0.0)	1 (6.7))	2 (9.1)
	Non-randomized controlled trial	41 (91.1)	5 (100.0)	3 (100.0)	13 (86.7))	20 (90.9)
	One-group pre-post study	1 (2.2)	0 (0.0)	0 (0.0)	1 (6.7)	0 (0.0)
Type of research^†^ (n=46)	Thesis or dissertation					
	Master	8 (17.4)	1 (20.0)	0 (0.0)	2 (13.3)	5 (21.7)
	Doctoral	4 (8.7)	1 (20.0)	0 (0.0)	0 (0.0)	3 (13.0)
	Funded research	9 (19.6)	1 (20.0)	1 (33.3)	2 (13.3)	5 (21.7)
	General research	25 (54.3)	2 (40.0)	2 (66.7)	11 (73.3)	10 (43.5)
Theoretical framework	Yes	11 (24.4)	1 (20.0)	1 (33.3)	1 (6.7)	8 (36.4)
	No	34 (75.6)	4 (80.0)	2 (66.7)	14 (93.3)	14 (63.6)
Intervention scope	Women	29 (64.4)	2 (40.0)	2 (66.7)	8 (53.3)	17 (77.3)
	Spouses	2 (4.4)	0 (0.0)	0 (0.0)	1 (6.7)	1 (4.6)
	Women+spouses	9 (20.0)	3 (60.0)	1 (33.3)	2 (13.3)	3 (13.6)
	Women+spouses+family	2 (4.4)	0 (0.0)	0 (0.0)	2 (13.3)	0 (0.0)
	Women+infants	3 (6.7)	0 (0.0)	0 (0.0)	2 (13.3)	1 (4.6)
Primipara/multipara	Primiparae	22 (48.9)	5 (100.0)	3 (100.0)	8 (53.3)	6 (27.3)
	Primiparae+multiparae	23 (51.1)	0 (0.0)	0 (0.0)	7 (46.7)	16 (72.7)
Pregnancy period	During pregnancy					
	First trimester	1 (2.2)	0 (0.0)	0 (0.0)	0 (0.0)	1 (4.6)
	First-second	3 (6.7)	0 (0.0)	0 (0.0)	1 (6.7)	2 (9.1)
	Second trimester	2 (4.4)	0 (0.0)	0 (0.0)	0 (0.0)	2 (9.1)
	Second-third	11 (24.4)	0 (0.0)	2 (66.7)	3 (20.0)	6 (27.3)
	Third trimester	16 (35.6)	5 (100.0)	0 (0.0)	5 (33.3)	6 (27.3)
	Unspecified	5 (11.1)	0 (0.0)	0 (0.0)	3 (20.0)	2 (9.1)
	During pregnancy+labor and birth	2 (4.4)	0 (0.0)	0 (0.0)	1 (6.7)	1 (4.6)
	During labor and birth	5 (11.1)	0 (0.0)	1 (33.3)	2 (13.3)	2 (9.1)
High-risk pregnancy factors	Obstetric factors, infertility	1 (2.2)	0 (0.0)	0 (0.0)	0 (0.0)	1 (4.6)
	Physical factors, advanced age	1 (2.2)	0 (0.0)	0 (0.0)	0 (0.0)	1 (4.6)
	Current pregnancy factors					
	GDM	3 (6.7)	0 (0.0)	0 (0.0)	0 (0.0)	3 (13.6)
	Preterm labor	4 (8.9)	0 (0.0)	0 (0.0)	1 (6.7)	3 (13.6)
	Hyperemesis	1 (2.2)	0 (0.0)	0 (0.0)	1 (6.7)	0 (0.0)
	Cesarean section	2 (4.4)	0 (0.0)	0 (0.0)	1 (6.7)	1 (4.6)
	Unspecified high-risk pregnancy	1 (2.2)	0 (0.0)	0 (0.0)	0 (0.0)	1 (4.6)
	Specific population, immigrant women	1 (2.2)	0 (0.0)	0 (0.0)	0 (0.0)	1 (4.6)
	None	31 (68.9)	5 (100.0)	3 (100.0)	12 (80.0)	11 (50.0)
Sample size of each group (or total)	<30 (or total <60)	22 (48.9)	2 (40.0)	1 (33.3)	9 (60.0)	10 (45.5)
	30–49 (or total 60–99)	14 (31.1)	1 (20.0)	1 (33.3)	4 (26.7)	8 (36.4)
	>50 (or total >100)	9 (20.0)	2 (40.0)	1 (33.3)	2 (13.3)	4 (18.2)

GDM: gestational diabetes mellitus.

† Duplicated answers.

**Table 2.** Characteristics of prenatal nursing interventions (N=45)

Variable	Categories	Total n(%)	1980–1989	1990–1999	2000–2009	2010–2019
			n=5	n=3	n=15	n=22
Study setting	Hospital	39 (86.7)	5 (100.0)	3 (100.0)	12 (80.0)	19 (86.4)
	Public health center	3 (6.7)	0 (0.0)	0 (0.0)	2 (13.3)	1 (4.6)
	Community	2 (4.4)	0 (0.0)	0 (0.0)	1 (6.7)	1 (4.6)
	Mobile web	1 (2.2)	0 (0.0)	0 (0.0)	0 (0.0)	1 (4.6)
Study unit	Group	18 (40.0)	4 (80.0)	2 (66.7)	7 (46.7)	5 (22.7)
	Individual	21 (46.7)	0 (0.0)	1 (33.3)	7 (46.7)	11 (50.0)
	Combination (group+individual)	5 (11.1)	1 (20.0)	0 (0.0)	1 (6.7)	5 (22.7)
	Other	1 (2.2)	0 (0.0)	0 (0.0)	0 (0.0)	1 (4.6)
Intervention methods^†^ (n=90)	Instruction	34 (37.8)	5 (62.5)	3 (50.0)	10 (34.5)	16 (34.0)
	Demonstration	11 (12.2)	0 (0.0)	0 (0.0)	4 (13.8)	7 (14.9)
	Practice	21 (23.3)	2 (25.0)	2 (33.3)	8 (27.6)	9 (19.2)
	Counseling	8 (8.9)	1 (12.5)	0 (0.0)	1 (3.5)	6 (12.8)
	Phone call	7 (7.8)	0 (0.0)	1 (16.7)	2 (6.9)	4 (8.5)
	Others	9 (10.0)	0 (0.0)	0 (0.0)	4 (13.8)	5 (10.6)
Experimental period	1 day	11 (24.4)	2 (40.0)	1 (33.3)	3 (20.0)	5 (22.7)
	2–6 days	7 (15.6)	0 (0.0)	0 (0.0)	3 (20.0)	4 (18.2)
	1–2 weeks	3 (6.7)	0 (0.0)	1 (33.3)	0 (0.0)	2 (9.1)
	3–6 weeks	18 (40.0)	3 (60.0)	1 (33.3)	7 (46.7)	7 (31.8)
	7–12 weeks	6 (13.3)	0 (0.0)	0 (0.0)	2 (13.3)	4 (18.2)
Number of sessions	1–4	20 (44.4)	2 (40.0)	1 (33.3)	8 (53.3)	9 (40.9)
	5–8	15 (33.3)	3 (60.0)	2 (66.7)	4 (26.7)	6 (27.3)
	9–12	5 (11.1)	0 (0.0)	0 (0.0)	0 (0.0)	5 (22.7)
	> 13	4 (8.9)	0 (0.0)	0 (0.0)	3 (20.0)	1 (4.6)
	Others	1 (2.2)	0 (0.0)	0 (0.0)	0 (0.0)	1 (4.6)
Time per session	0–60 minutes	24 (53.3)	3 (60.0)	1 (33.3)	6 (40.0)	14 (63.6)
	61–120 minutes	13 (28.9)	2 (40.0)	0 (0.0)	5 (33.3)	6 (27.3)
	Over 2 hours	2 (4.4)	0 (0.0)	1 (33.3)	1 (6.7)	0 (0.0)
	Not described	6 (13.3)	0 (0.0)	1 (33.3)	3 (20.0)	2 (9.1)
Follow-up period	Immediately after intervention	19 (42.2)	0 (0.0)	0 (0.0)	6 (40.0)	13 (59.1)
	1–6 days after intervention	1 (2.2)	0 (0.0)	0 (0.0)	1 (6.7)	0 (0.0)
	1–4 weeks after intervention	3 (6.7)	2 (40.0)	0 (0.0)	0 (0.0)	1 (4.6)
	8 weeks after intervention	1 (2.2)	0 (0.0)	0 (0.0)	0 (0.0)	1 (4.6)
	Post–delivery, 0–6 days	17 (37.8)	3 (60.0)	2 (66.7)	7 (46.7)	5 (22.7)
	Post–delivery, 1–4 weeks	2 (4.4)	0 (0.0)	0 (0.0)	1 (6.7)	1 (4.6)
	Post–delivery, 5–8 weeks	1 (2.2)	0 (0.0)	1 (33.3)	0 (0.0)	0 (0.0)
	Post–delivery, 9–12 weeks	1 (2.2)	0 (0.0)	0 (0.0)	0 (0.0)	1 (4.6)
Intervention type	Prenatal health education	22 (48.9)	4 (80.0)	3 (100.0)	4 (26.7)	11 (50.0)
	Pregnancy health care and childbirth	12 (26.7)	4 (80.0)	2 (66.7)	3 (20.0)	3 (13.6)
	Breastfeeding and breast care	2 (4.4)	0 (0.0)	1 (33.3)	0 (0.0)	1 (4.6)
	Newborn care and parental role	3 (6.7)	0 (0.0)	0 (0.0)	1 (6.7)	2 (9.1)
	High-risk pregnancy health care	4 (8.9)	0 (0.0)	0 (0.0)	0 (0.0)	4 (18.2)
	Health-related behavior	1 (2.2)	0 (0.0)	0 (0.0)	0 (0.0)	1 (4.6)
	Psychosocial support	6 (13.3)	1 (20.0)	0 (0.0)	3 (20.0)	2 (9.1)
	Family participated delivery	3 (6.7)	0 (0.0)	0 (0.0)	2 (13.3)	1 (4.6)
	Spouse support reinforcement	3 (6.7)	1 (20.0)	0 (0.0)	1 (6.7)	1 (4.6)
	Complementary therapy	17 (37.8)	0 (0.0)	0 (0.0)	8 (53.3)	9 (40.9)
	Relaxation and abdominal breathing	4 (8.9)	0 (0.0)	0 (0.0)	2 (13.3)	2 (9.1)
	Music therapy	4 (8.9)	0 (0.0)	0 (0.0)	1 (6.7)	3 (13.6)
	Yoga and qigong training	4 (8.9)	0 (0.0)	0 (0.0)	3 (20.0)	1 (4.6)
	Therapeutic touch and massage	2 (4.4)	0 (0.0)	0 (0.0)	0 (0.0)	2 (9.1)
	Acupressure	2 (4.4)	0 (0.0)	0 (0.0)	2 (13.3)	0 (0.0)
	Aroma inhalation	1 (2.2)	0 (0.0)	0 (0.0)	0 (0.0)	1 (4.6)

† Duplicated answers.

**Table 3.** Types of outcome variables (N=128)

Variable		1980–1989	1990–1999	2000–2009	2010–2019
Psychological		Anxiety (3)	Anxiety (1)	Anxiety (7)	Anxiety (10)
		Antenatal		Antenatal stress (1)	Depression (5)
		stress (1)		Depression (1)	Stress (antenatal, 5; preterm labor, 3; child rearing, 1; perceived, 1)
		Spouse support (1)		Self-confidence (maternal role) (1)	Self-efficacy (childbirth, 2; child rearing, 1; GDM, 1; pregnancy health care, 1)
				Spouse support (1)	Self-confidence (childbirth, 3; maternal role, 1; paternal role, 1)
				Spouse participation (2)	Quality of life (1)
					Spouse support (1)
					Maternal identity (1)
Interpersonal		Mother-infant synchrony (1)		Maternal-fetal attachment (1)	Maternal-fetal attachment (2)
		Infant response (1)		Mother-infant interaction (1)	Father-infant attachment (2)
					Couple attachment (1)
Perceptual		Maternal sensitivity (1)		Knowledge (childbirth, 1; breastfeeding, 1)	Knowledge (child rearing, 1; GDM, 1; breastfeeding, 1; pregnancy health care, 1)
				Perception of childbirth experience (3)	Perception of childbirth experience (2)
Behavioral		Maternal attitude of childrearing (1)	Breastfeeding practice (1)		Breastfeeding methods (1)
					Breastfeeding rate (1)
					GDM self-management (1)
					Oral health behavior (1)
					Pregnancy health care practice behavior (1)
					Self-care behavior (GDM) (1)
Physiological		Labor pain (1)	Labor pain (2)	Labor pain (5)	Labor pain (1)
		Length of labor (1)	Pulse (1)	Length of labor (2)	Vital signs (2)
		Apgar score (1)	Breast discomfort (1)	Vital signs (2)	Physical activity (1)
				Pulse (1)	Periodontal disease (1)
				S_a_O_2 _(1)	Uterine contractions (1)
				Weight gain (1)	Tocolytic dosage (1)
				Vomiting (1)	Biochemical index (2)
				Discomfort (nausea/vomiting, 1; after childbirth, 1)	ANS (1)
				Physical symptoms (1)	Maternal sleep/activity (1)
				Fatigue (1)	Infant's sleep/activity (1)
				Cortisol level (1)	FHR (2)
				Infant (BW, 2; HC, 1)	
Total, n (%)	128 (100.0)	12 (9.4)	6 (4.7)	41 (32.0)	69 (53.9)

ANS: autonomic nervous system; BW: body weight; FHR: fetal heart rate; GDM: gestational diabetes mellitus; HC: head circumference; S_a_O_2_: oxygen saturation.

## Supplementary materials

Further details on supplementary materials are presented online (available at https://doi.org/10.4069/kjwhn.2020.06.12).

Supplement 1. List of selected literature.

Supplement 2. Literature review findings.
