# Supplementary material for: Prenatal nursing intervention studies published in Korean nursing journals: a scoping review
Source: Korean J Women Health Nurs. 2020 Jun 22;26(2):109–19. doi: 10.4069/kjwhn.2020.06.12 (PMC9328601; doi:10.4069/kjwhn.2020.06.12)
Supplement: Supplement 1. — List of selected literature. [file kjwhn-2020-06-12suppl1.pdf]

Supplement 1 List of selected literature (n=45)

S1. Kim YS, Lee YJ, Park GH. The effects of maternal health care program on depression, maternal role self-confidence and delivery self-efficacy in pregnant women. J East-West Nurs Res. 2017;23(2):134-141. https://doi.org/10.14370/jewnr.2017.23.2.134

S2. Kang YS, Jun MH, Kim IS. Study of influence on the antenatal instruction according to the state-anxiety level in normal process of primigravida. J Ewha Nurs Sci. 1981;15(1):9-13.

S3. Moon YS. A study on antenatal education about the normal course of labor in last trimester of gestation influencing the state: anxiety of primigravida. J Nurs Acad Soc. 1980;10(1):11-20. https://doi.org/10.4040/jnas.1980.10.1.11

S4. Kim S, Kim HS, Cheong HY. Effects of a coaching-based childbirth program on anxiety and childbirth self-efficacy among primigravida women. Korean J Women Health Nurs. 2011;17(4):369-377. http://dx.doi.org/10.4069/kjwhn.2011.17.4.369

S5. Park M, Lee S. Effects of an experience-focused prenatal program on stress, anxiety, childbirth confidence, and maternal-fetal attachment on women in their first pregnancy. Korean J Women Health Nurs. 2018;24(2):126-137. https://doi.org/10.4069/kjwhn.2018.24.2.126

S6. Sung MH, Ju MS, Ju KS. Effects of a prepared childbirth education on the knowledge and delivery participation levels of the spouses of primiparas. Korean J Women Health Nurs. 2003;9(3):213-223. https://doi.org/10.4069/kjwhn.2003.9.3.213

S7. Kim S, Kim H, Cho S, Kim O. The effects of childbirth education on labor pain, anxiety and perception of childbirth experience of primiparas. Clin Nurs Res. 2002;8(1):129-145.

S8. Lee EL, Lee HJ, Oh JH, Kim SA. The effect of a Lamaze program on labor pain in primiparas. Clin Nurs Res. 1998;4(1):59-84.

S9. Kim DH, Kim SJ, Kim YS, Kim YK, Park EY, Bae KE, et al. Effects of breathing training education on labor pain and anxiety during labor in latent phase for primigravida. J Ewha Nurs Sci. 1995;(28):1-10.

S10. Park YS. A study on the evaluation of Lamaze childbirth educational program. J Nurs Acad Soc. 1987;17(1):52-63. https://doi.org/10.4040/jnas.1987.17.1.52

S11. Han KJ, Park YS. A study on the effects of the Lamaze prophylaxis. J Nurs Acad Soc. 1985;15(2):16-24. https://doi.org/10.4040/jnas.1985.15.2.16

S12. Kim JS, Kim MS, Kim YH, Kim YS, Kim BJ, Lee JH. The effects of sophrologic prenatal education program in community health centers. J Korean Community Nurs. 2003;14(4):598-607.

S13. Kim JK, Choi HM, Ryu EJ. Postnatal breast-feeding knowledge, techniques and rates of first-time mothers depending on a prenatal breast-feeding education method. J Korean Acad Soc Nurs Educ. 2011;17(1):90-99. https://doi.org/10.5977/JKASNE.2011.17.1.090

S14. Park OH. The effects of antenaltal breast care on breast feeding practice. J Nurs Acad Soc. 1992;22(1):29-41. https://doi.org/10.4040/jnas.1992.22.1.29

S15. Lee GM, Choi YH. Effects of newborn care education program on child-rearing knowledge, child-rearing stress, and child-rearing self-efficacy of immigrant pregnant women. J Korean Public Health Nurs. 2012;26(3):561-571. https://doi.org/10.5932/JKPHN.2012.26.3.561

S16. Han KJ, Kwon MK, Bang KS, Choi MY. Effects of a mother-fetus interaction promotion program on sleep and activity in mothers and infants. J Korean Acad Child Health Nurs. 2010;16(4):239-248. https://doi.org/10.4094/jkachn.2010.16.4.239

S17. Kim TI. Development and test of effectiveness of a prenatal parental role education program. J Korean Acad Child Health Nurs. 2006;12(1):104-113.

S18. Ko JM, Lee JK. Effects of a coaching program on comprehensive lifestyle modification for women with gestational diabetes mellitus. J Korean Acad Nurs. 2014;44(6):672-681. https://doi.org/10.4040/jkan.2014.44.6.672

S19. Kim H, Kim S. Effects of an integrated self-management program on self-management, glycemic control, and maternal identity in women with gestational diabetes mellitus. J Korean Acad Nurs. 2013;43(1):69-80. https://doi.org/10.4040/jkan.2013.43.1.69

S20. Baek ES, Park HJ. Effects of a case management program on self-efficacy, depression and anxiety in pregnant women with gestational diabetes mellitus. Korean J Women Health Nurs. 2013;19(2):88-98. https://doi.org/10.4069/kjwhn.2013.19.2.88

S21. Wang HJ, Kim IO. Effects of a mobile web-based pregnancy health care educational program for mothers at an advanced maternal age. J Korean Acad Nurs. 2015;45(3):337-346. https://doi.org/10.4040/jkan.2015.45.3.337

S22. Park H, Lee HJ. Comparison of effects of oral health program and walking exercise program on health outcomes for pregnant

women. J Korean Acad Nurs. 2018;48(5):506-520. https://doi.org/10.4040/jkan.2018.48.5.506
S23. Yu WJ, Song JE. Effects of abdominal breathing on state anxiety, stress, and tocolytic dosage for pregnant women in preterm labor. J Korean Acad Nurs. 2010;40(3):442-452. https://doi.org/10.4040/jkan.2010.40.3.442
S24. Chang SB, Kim HS, Ko YH, Bae CH, An SE. Effects of abdominal breathing on anxiety, blood pressure, peripheral skin temperature and saturation oxygen of pregnant women in preterm labor. Korean J Women Health Nurs. 2009;15(1):32-42. http://dx.doi.org/10.4069/kjwhn.2009.15.1.32
S25. Choi MS, Park YJ. The effects of relaxation therapy on anxiety and stress of pregnant women with preterm labor. Korean J Women Health Nurs. 2010;16(4):336-347. http://dx.doi.org/10.4069/kjwhn.2010.16.4.336
S26. An SE. Effects of abdominal breathing on anxiety and labor time in primipara women. Korean J Women Health Nurs. 2008;14(3):196-204. https://doi.org/10.4069/kjwhn.2008.14.3.196
S27. Oh MO, Kim YJ, Baek CH, Kim JH, Park NM, Yu MJ, et al. Effect of music intervention on maternal anxiety and fetal heart rate pattern during non-stress test. J Korean Acad Nurs. 2016;46(3):315-326. https://doi.org/10.4040/jkan.2016.46.3.315
S28. Shin HS, Kim JH. Music therapy on anxiety, stress and maternal-fetal attachment in pregnant women during transvaginal ultrasound. Asian Nurs Res. 2011;5(1):19-27. https://doi.org/10.1016/S1976-1317(11)60010-8
S29. Park HJ, Sung MH. Effects of music therapy on stress of preterm labor and uterine contraction in pregnant women with preterm labor. Korean J Women Health Nurs. 2017;23(2):109-116. https://doi.org/10.4069/kjwhn.2017.23.2.109
S30. Kwun YS, Kim TH. The effect of music therapy on anxiety of cesarean section wemen. J Korean Acad Fundam Nurs. 2000;7(3):466-478.
S31. Ji ES, Cho KJ, Kwon HJ. Effects of yoga during pregnancy on weight gain, delivery experience and infant's birth weight. Korean J Women Health Nurs. 2009;15(2):121-129. http://dx.doi.org/10.4069/kjwhn.2009.15.2.121
S32. Shim CS, Lee YS. Effects of a yoga-focused prenatal program on stress, anxiety, self confidence and labor pain in pregnant women with in vitro fertilization treatment. J Korean Acad Nurs. 2012;42(3):369-376. http://dx.doi.org/10.4040/jkan.2012.42.3.369
S33. Jeong SO, Kho Hj, Lee EJ. Effects of a Qigong training program on the anxiety and labor pain of primipara. Korean J Women Health Nurs. 2006;12(2):97-105. https://doi.org/10.4069/kjwhn.2006.12.2.97
S34. Lee KO, Kim KR, Ahn SH. Effects of a Qigong prenatal education program on anxiety, depression and physical symptoms in pregnant women. Korean J Women Health Nurs. 2006;12(3):240-248. https://doi.org/10.4069/kjwhn.2006.12.3.240
S35. Choi MS, Kim GJ. Effects of paters' duola touch during labor on the paternal attachment and role confidence to neonate and couple attachment. Korean J Women Health Nurs. 2011;17(4):426-437. http://dx.doi.org/10.4069/kjwhn.2011.17.4.426
S36. Jeon SI, Park MK. Effects of a spouse's hand massage on anxiety, spouse support and the perception on childbirth experience of a primipara with analgesia for vaginal delivery. J Korean Soc Matern Child Health. 2015;19(2):233-242. https://doi.org/10.21896/jksmch.2015.19.2.233
S37. Shin HS, Song YA, Hwang JI. Effects of hand stimulation intervention on fatigue and the blood cortisol level of pregnant women. J Korean Acad Soc Nurs Educ. 2007;13(2):212-219.
S38. Shin HS, Song YA. The effect of P6 acupressure for symptom control in pregnant women having hyperemesis gravidarum. J Korean Acad Nurs. 2005;35(3):593-601. https://doi.org/10.4040/jkan.2005.35.3.593
S39. Go GY, Park H. Effects of aroma inhalation therapy on stress, anxiety, depression, and the autonomic nervous system in high-risk pregnant women. Korean J Women Health Nurs. 2017;23(1):33-41. https://doi.org/10.4069/kjwhn.2017.23.1.33
S40. Eom SY, Kim ES, Kim HJ, Bang YO, Chun N. Effects of a one session spouse-support enhancement childbirth education on childbirth self-efficacy and perception of childbirth experience in women and their husbands. J Korean Acad Nurs. 2012;42(4):599-607. https://doi.org/10.4040/jkan.2012.42.4.599
S41. Kwak IJ, Park KH, Lee MY, Kim MR, Lee HS, Hong YM, et al. The effect of one session spouse's support reinforcement prenatal education program to participation and support degree of spouse to pregnant women during labor. Clin Nurs Res. 2004;10(1):42-55.
S42. Ahn HL. An experimental study of the effects of husband's supportive behavior reinforcement education on stress relief of primigravidas. J Nurs Acad Soc. 1985;15(1):5-16. https://doi.org/10.4040/jnas.1985.15.1.5
S43. Oh JH, Lee HJ, Kim YK, Min J, Park KO. The effect of childbirth education and family participated delivery in a labor-delivery-recovery room on primiparas' anxiety, labor pain and perception of childbirth experience. Clin Nurs Res. 2006;12(2):145-

156.
S44. Jang MJ, Park KS. Effect of family-participated delivery in a labor delivery room on the childbirth of primiparas. Korean J Women Health Nurs. 2002;8(3):371-379. https://doi.org/10.4069/kjwhn.2002.8.3.371
S45. Choi MR, Kim HK. Effects of a paternal participation program during cesarean section on paternal infant attachment. Korean J Women Health Nurs. 2013;19(2):75-87. https://dx.doi.org/10.4069/kjwhn.2013.19.2.75
