# Supplementary material for: Prenatal nursing intervention studies published in Korean nursing journals: a scoping review
Source: Korean J Women Health Nurs. 2020 Jun 22;26(2):109–19. doi: 10.4069/kjwhn.2020.06.12 (PMC9328601; doi:10.4069/kjwhn.2020.06.12)
Supplement: Supplement 2. — Literature review findings. [file kjwhn-2020-06-12suppl2.pdf]

Supplement 2 Literature review findings (N=45)

Author (year)	Research design	Theoretical framework	Study participants: Pregnancy period (if primipara)/ High-risk factors	Study participants: Sample size	Intervention: Scope	Intervention: Setting /unit	Intervention: Name of intervention (contents)	Intervention: Methods (tools)	Intervention: Experimental period	Intervention: Sessions	Intervention: Time per session (minute)	Follow-up period	Outcome variables
I.	Prenatal health education (n = 22)												
I-1. Prenatal care & childbirth (n = 12)													
Kim et al. [S1] (2017)	NRCT	NA	During pregnancy/ None	E:39 C:40	Women	Public health center /group	Maternal health care program (nutrition management, Lamaze breathing, newborn care, postpartum, care, breastfeeding communication with spouse for prevention of prenatal or postnatal depression, yoga)	Instruction, demonstration, and practice (video)	8 wks	8	120	Immediately after intervention	Depression (–), self-confidence (maternal role) (+), self-confidence (childbirth) (+)
Kang et al. [S2] (1981)	NRCT	NA	During pregnancy (IUP over 32 wks) (primipa-ra)/None	E:40 C:40	Women	Hospital /individual +group	Prenatal education about normal course of labor (physiology and progress of labor and delivery, hygiene during pregnancy, preparation of childbirth)	Instruction and counseling	1 day	1	15–20	During labor (latent phase)	State anxiety (+)
Moon [S3] (1980)	NRCT	NA	During pregnancy (IUP over 36 wks) (primipara) /None	E:75 C:75	Women	Hospital /group	Prenatal education about normal course of labor (symptoms when birth is near, time to be hospitalized, progress of delivery and labor, breathing method)	Instruction	1 day	1	40	1 wk after intervention	State anxiety (+)
Kim et al. [S4] (2011)	NRCT	GROW Coach-ing Model (Whitmore, 2008)	During pregnancy (IUP over 28 wks) (primipara) /None	E:29 C:31	Women	Hospital /group	Coaching-based childbirth program (pregnancy adaptation with fetal growth, normal delivery, emergency management, emotional change, postpartum care, newborn care, preparation for parenthood)	Instruction and counseling	4 wks	4	120	Immediately after intervention	State anxiety (–), self-efficacy (childbirth) (+)
Park and Lee [S5] (2018)	NRCT	NA	During pregnancy (IUP over 32 wks) (primipara) /None	E:28 C:29	Women	Hospital /individual +group	Experience-focused prenatal program (overall understanding of normal pregnancy, normal delivery, newborn care, breastfeeding)	Instruction, practice, counseling, discussion and tour (PPT, video, and model)	4 wks	4	100	Immediately after intervention	Antenatal stress (+), state anxiety (+), self- confidence (childbirth) (+), maternal-fetal attachment (+)

(Continued to the next page)

Author (year)	Research design	Theoretical framework	Study participants: Pregnancy period (if primipara)/ High-risk factors	Study participants: Sample size	Intervention: Scope	Intervention: Setting /unit	Intervention: Name of intervention (contents)	Intervention: Methods (tools)	Intervention: Experimental period	Intervention: Sessions	Intervention: Time per session (minute)	Follow-up period	Outcome variables
Sung et al. [S6] (2003)	NRCT	NA	During pregnancy (IUP 37–42 wks) (primipara) /None	E:74 C:52	Spouses	Hospital /group	Prepared childbirth education (Lamaze method, physiology and anatomy of pregnancy, labor, childbirth, normal versus cesarean delivery, newborn care, breastfeeding, postpartum care, family planning)	Instruction and practice (booklet, video, OHP, model)	5 wks	5	120	< 2 hours post-delivery	Knowledge (childbirth) (+), spouse participation (+)
Kim et al. [S7] (2002)	NRCT	NA	During pregnancy (IUP 28–32 wks) (primipara) /None	E:25 C:33	Women and spouses	Hospital /group	Childbirth education (physiology and anatomy of pregnancy, antenatal care, Lamaze method, breathing, massage, relaxation, imagery, process of delivery, postpartum care, breastfeeding, Kegel exercises)	Instruction	4 wks	4	150	1 day post-delivery	State anxiety (–), labor pain (+), pulse (–), perceptions of delivery experience (+)
Lee et al. [S8] (1998)	NRCT	Gate control theory of pain (Melzack & Casey, 1968)	During pregnancy (IUP 22–32 wks) (primipara)/ None	E:19 C:20	Women and spouse	Hospital /group	Lamaze program (physiology and anatomy of pregnancy, process of delivery, nutrition, gymnastics, Lamaze method, breathing, relaxation, newborn care, breastfeeding)	Instruction (video)	6 wks	6	180	1 hour post-delivery	Labor pain (+), pulse (–)
Kim et al. [S9] (1995)	NRCT	NA	During labor and birth (IUP 38–42 wks) (primipara)/ None	E:35 C:35	Women	Hospital /individual	Breathing training education during childbirth in latent phase (Lamaze breathing, physiology, and process of labor and delivery)	Instruction and practice	1 day	5	Unclear	During labor (latent phase)	State anxiety (+), labor pain (+)
Park [S10] (1987)	NRCT	Research framework (developed by researcher)	During pregnancy (IUP 28–32 wks) (primipara)/ None	E:10 C:10	Women and spouse	Hospital /group	Lamaze method education (Lamaze breathing and relaxation)	Instruction and practice	6 wks	6	120	During labor (latent, active, transition phase)	State anxiety (+), labor pain (–), length of labor (+), Apgar score (–)
Han and Park [S11] (1985)	NRCT	NA	During pregnancy (IUP 28–32 wks) (primipara)/None	E:9 C:10	Women and spouses	Hospital /group	Lamaze prophylaxis education (awareness of pregnant women herself, breathing training, expectations of childbirth and infant abilities)	Instruction and practice	5 wks	5	120	2-3 days post-delivery	Maternal attitude (childrearing) (–), maternal sensitivity (+), infant response (+), mother-infant dyadic synchrony (–)

(Continued to the next page)

Author (year)	Research design	Theoretical framework	Study participants: Pregnancy period (if primipara)/High-risk factors	Study participants: Sample size	Intervention: Scope	Intervention: Setting /unit	Intervention: Name of intervention (contents)	Intervention: Methods (tools)	Intervention: Experimental period	Intervention: Sessions	Intervention: Time per session (minute)	Follow-up period	Outcome variables
Kim et al. [S12] (2003)	One group pre-post study	NA	During pregnancy/None	16	Women	Public health center/group	Sophrologic prenatal education programs (sophrologic method, relaxation, breathing, focusing, physiology and anatomy of childbirth, process of delivery, spouse support, breastfeeding, childrearing, postpartum care)	Instruction and practice	8 wks	8	Unclear	Immediately after intervention	Knowledge (breastfeeding) (+), maternal-fetal attachment (–), antenatal stress(–)
I-2. Breastfeeding and breast care (n = 2)													
Kim et al. [S13] (2011)	NRCT	NA	During pregnancy (IUP over 35 wks) (primipara) /None	E:70 C:69	Women	Hospital /individual	Prenatal breastfeeding education (breast assessment, breast care and massage, breastfeeding methods, postpartum breastfeeding problems)	Instruction, demonstration and practice (booklet, video, PPT, model)	2-4 wks	2	25	12 wks post-delivery	Breastfeeding methods (+), breastfeeding rate (+), knowledge (breastfeeding) (–)
Park [S14] (1992)	NRCT	NA	During pregnancy (IUP 20–30 wks) (primipara) /None	E:51 C:75	Women	Hospital /group	Prenatal breast care (breastfeeding methods and practice, breast care)	Instruction, practice and follow-up call	1-2 wks	2	60	2 months post-delivery	Breastfeeding practice (+), breast discomfort (+)
I-3. Newborn care and parental role (n = 3)													
Lee and Choi [S15] (2012)	NRCT	Self-efficacy theory (Bandura, 1977)	During pregnancy/ Immigrant women	E:17 C:16	Women	Community center /group	Newborn care education program (physical and psychological characteristics of newborn, bathing, umbilical care, feeding, trimming, diapering, checking temperature, detection adverse symptom and care)	Instruction, demonstration and practice (booklet, video, PPT, model)	3 wks	3	90	Immediately after intervention	Knowledge (newborn care) (+), stress (+), self-efficacy (childrearing) (+)
Han et al. [S16] (2010)	NRCT	NA	During pregnancy (IUP 31–38 wks) (primipara) /None	E:17 C:17	Women and infants	Hospital /group	Mother-fetus interaction promotion program (taekyo, importance of mother-fetus interaction, maternal role education)	Instruction and discussion (booklet, PPT, sleep/activity record)	8 wks	8	60	4 wks post-delivery	Maternal sleep/ activity (–), infant's sleep/ activity (–)

(Continued to the next page)

Author (year)	Research design	Theoretical framework	Study participants: Pregnancy period (if primipara)/High-risk factors	Study participants: Sample size	Intervention: Scope	Intervention: Setting/unit	Intervention: Name of intervention (contents)	Intervention: Methods (tools)	Intervention: Experimental period	Intervention: Sessions	Intervention: Time per session (minute)	Follow-up period	Outcome variables
Kim [S17] (2006)	NRCT	NA	During pregnancy (IUP over 32 wks) (primipara) /None	E:27 C:30	Women and infants	Hospital /group	Prenatal Parental Role Education Program (parental role education, infant care, mother-infant interaction, breastfeeding, integrated sensory stimulation)	Instruction, practice and phone counseling (booklet, video, model)	4 wks	4 (+2 by phone call)	Unclear	4 wks post-delivery	Self-confidence (maternal role) (+), mother-infant interaction (+), infant BW (+), HC (+)
I-4. High-risk pregnancy health care (n = 4)													
Ko and Lee [S18] (2014)	NRCT	GROW Coaching Model (Whitmore, 2008)	During pregnancy (IUP 24 wks)/GDM	E:34 C:34	Women	Hospital /individual +group	Coaching Program on Comprehensive Lifestyle Modification for Women with GDM (glycemic control, diet and breastfeeding, exercise, delivery method, postpartum care)	Instruction, counseling and phone counseling (booklet, PPT, CD, diary)	4 wks	4 (+4 by phone)	60 (20 by phone)	8 wks after intervention	Knowledge (GDM) (–), self-care behavior (GDM) (+), depression (+), FBS (+), HbA1C (+)
Kim and Kim [S19] (2013)	NRCT	IMCHB (Cox, 1982)	During pregnancy (IUP 24–30 wks)/GDM	E:28 C:27	Women	Hospital /individual +group	Integrated Self-Management Program for Women with GDM (diet, exercise, stress management, self-checking and management of blood glucose, abdominal breathing, maternal role, maternal-fetal interaction, emotional reactions, postpartum care)	Instruction and phone counseling (booklet)	4 wks	3 (+2 by phone)	60 (10–15 by phone)	2 wks after intervention	Self-management (GDM) (+), PP2 (+), HbA1c (–), maternal identity (+)
Baek and Park [S20] (2013)	NRCT	Self-efficacy theory (Bandura, 1977)	During pregnancy (IUP 24–28 wks)/GDM	E:19 C:18	Women	Hospital /individual	Case Management Program for Women with GDM (self-monitoring of blood glucose, diet, exercise, postpartum care, emotional reactions, incidence of type 2 diabetes, importance of goal setting and action of glycemic control, motivation and behavior change)	Counseling and phone counseling (booklet, diary)	2 wks	1 (+5 by phone)	Unclear	Immediately after intervention	Self-efficacy (GDM) (+), depression (+), anxiety (+)

(Continued to the next page)

Author (year)	Research design	Theoretical framework	Study participants: Pregnancy period (if primipara)/ High-risk factors	Study participants: Sample size	Intervention: Scope	Intervention: Setting /unit	Intervention: Name of intervention (contents)	Intervention: Methods (tools)	Intervention: Experimental period	Intervention: Sessions	Intervention: Time per session (minute)	Follow-up period	Outcome variables
Wang and Kim [S21] (2015)	NRCT	Web-based teaching -learning system design	During pregnancy (IUP < 20 wks)/ Advanced age	E:30 C:31	Women	Mobile website	Mobile web-based pregnancy health care education for mothers at an advanced maternal age (understanding pregnancy at advanced age, fetal development, symptom control, health problems control, nutrition management, daily life, exercise, antenatal testing, emotional change, working life)	Instruction (mobile web site)	2 wks	-	-	Immediately after intervention	Knowledge (+), self-efficacy (+), pregnancy health care practice behavior (pregnancy health care) (+)
I-5. Health-related behavior (n = 1)													
Park and Lee [S22] (2018)	RCT	IMCHB Cox (1982)	During pregnancy (IUP 16–24 wks) (primipara) /None	E1:23 E2:21 C:21	Women	Hospital /individual +group	Oral health program and walking exercise program (oral health care and dietary management, walking exercise)	Instruction, demonstration, practice, counseling, phone counseling and text messages (booklet, video, diary, model, pictures)	12 wks	3 (+9 by phone)	20–40	Immediately after intervention	Physical activity (+), periodontal disease (+), oral health behavior (+), antenatal stress (+), perceived stress (+), depression (+), quality of life (+)
II. Complementary Therapy (n = 17)													
II-1. Relaxation and abdominal breathing (n = 4)													
Yu and Song [S23] (2010)	NRCT	Research framework (developed by researcher)	During pregnancy (IUP 20–37 wks)/ Preterm labor	E:30 C:30	Women	Hospital /individual	Abdominal breathing for pregnant women with preterm labor	Instruction and practice	3 days	9	1.6	Immediately after intervention	State anxiety (+), preterm labor stress (+), tocolytic dosage (+)
Chang et al. [S24] (2009)	NRCT	Research framework (developed by researcher)	During pregnancy (IUP 24–37 wks)/ Preterm labor	E:26 C:20	Women	Hospital /individual	Abdominal breathing for pregnant women with preterm labor	Instruction and practice	3 days	3 (30 times/ session)	5	Immediately after intervention	State anxiety (+), BP, BT (+), S_aO_2 (+)

(Continued to the next page)

Author (year)	Research design	Theoretical framework	Study participants: Pregnancy period (if primipara)/High-risk factors	Study participants: Sample size	Intervention: Scope	Intervention: Setting /unit	Intervention: Name of intervention (contents)	Intervention: Methods (tools)	Intervention: Experimental period	Intervention: Sessions	Intervention: Time per session (minute)	Follow-up period	Outcome variables
Choi and Park [S25] (2010)	NRCT	NA	During pregnancy (IUP 20–38 wks)/ Preterm labor	E:29 C:26	Women	Hospital /individual	Relaxation therapy for pregnant women with preterm labor	Listening to a CD	5 days	10	7	Immediately after intervention	Preterm labor stress (+), state anxiety (+), BP, BT, pulse (+), FHR (–)
Ahn [S26] (2008)	NRCT	NA	During labor and birth (IUP 37–42 wks) (primipara) /None	E:15 C:21	Women	Hospital /individual	Abdominal breathing during labor	Instruction and practice	1 day	2 (30 times/ session)	5	Immediately after intervention	State anxiety (+), length of labor (–)
II-2. Music Therapy (n = 4)													
Oh et al. [S27] (2016)	RCT (cross over)	NA	During pregnancy (IUP 28-40 wks)/None	E:30 C:30	Women	Hospital /individual	Music intervention during non-stress test	Listening to music	1 day	2	20	Immediately after intervention	State anxiety (+), BP, HR, BT (–), FHR (+)
Shin and Kim [S28] (2011)	NRCT	NA	During pregnancy (IUP before 14 wks)/ None	E:117 C:116	Women	Hospital /individual	Music therapy during transvaginal ultrasound	Listening to music	1 day	1	30	Immediately after intervention	State anxiety (+), antenatal stress (–), maternal-fetal attachment (–)
Park and Sung [S29] (2017)	NRCT	NA	During pregnancy (IUP 20–37 wks)/ Preterm labor	E:17 C:18	Women	Hospital /individual	Music therapy for pregnant women with preterm labor	Listening to music	4 days	12	15	Immediately after intervention	Preterm labor stress (+), frequency of uterine contraction (–)
Kwun and Kim [S30] (2000)	NRCT	NA	During pregnancy/ Cesarean section	E:33 C:32	Women	Hospital /individual	Music therapy for women undergoing a cesarean section	Listening to music	2 days	3	30	Immediately after intervention	State anxiety (+), BP, pulse, RR (+)
II-3. Yoga and Qigong Training (n = 4)													
Ji et al. [S31] (2009)	NRCT	NA	During pregnancy (IUP over 20 wks)/ None	E:21 C:20	Women + infants	Community center/ group	Prenatal yoga	Instruction, demonstration, practice, and follow-up call	12 wks	24	90	Postpartum	BMI (+), labor pain (+), discomfort after delivery (+), infant's BW (+)

(Continued to the next page)

Author (year)	Research design	Theoretical framework	Study participants: Pregnancy period (if primipara)/ High-risk factors	Study participants: Sample size	Intervention: Scope	Intervention: Setting /unit	Intervention: Name of intervention (contents)	Intervention: Methods (tools)	Intervention: Experimental period	Intervention: Sessions	Intervention: Time per session (minute)	Follow-up period	Outcome variables
Shim and Lee [S32] (2012)	NRCT	NA	During pregnancy (IUP 12–20 wks)/ Pregnant with IVF	E:23 C:23	Women	Hospital /group	Yoga-focused prenatal program in pregnant women with in vitro fertilization treatment (taekyo, Lamaze, breastfeeding, process of delivery, newborn care, yoga)	Yoga and instruction (CD)	12 wks	12	75–80	During labor	Antenatal stress (+), state anxiety (+), self-confidence (childbirth) (+), labor pain (+)
Jeong et al. [S33] (2006)	NRCT	NA	During pregnancy (IUP 32–36 wks) (primipara) /None	E:30 C:30	Women	Hospital /group	Qigong training program	Demonstration and practice	4 wks	8	60	< 1 hour after delivery	State anxiety (+), labor pain (–)
Lee et al. [S34] (2006)	NRCT	NA	During pregnancy/ None	E:20 C:20	Women	Hospital /group	Qigong prenatal education program (definition, function, effects, nutrients, music, lifestyle of qigong taekyo, qigong training and meditation)	Instruction, demonstration, and practice	4 wks	4	120	Immediately after intervention	State anxiety (–), depression (+), physical symptoms (–)
II-4. Therapeutic touch and massage (n = 2)													
Choi and Kim [S35] (2011)	NRCT	NA	During pregnancy & labor and birth (IUP 32–42 wks)/None	E:60 C:60	Women and spouses	Hospital /individual +group	Paters' doula touch education (taekyo, fetus massage. normal process of delivery, doula touch, aroma massage, breathing method, newborn care, and breastfeeding) Paters' doula touch during labor	Instruction, demonstration and practice (booklet, video)	4 wks	4	120	8 hours post-delivery	Couple attachment (+), paternal attachment (–), paternal role confidence (+)
Jeon and Park [S36] (2015)	NRCT	NA	During labor and birth (IUP 37–42 wks) (primipara) /None	E:25 C:25	Women and spouses	Hospital /individual	Spouse's hand massage of women with analgesia for vaginal delivery	Instruction, demonstration and practice	1 day	04–06	10	2 hours post-delivery	State anxiety (+), spouse support (+), perception delivery experience (+)
II-5. Acupressure (n = 2)													
Shin et al. [S37] (2007)	NRCT	NA	During pregnancy (IUP 18–35 wks)/None	E:16 C:13	Women	Public health center /individual	Hand stimulation intervention	Acupressure	5 wks	15	40	Immediately after intervention	Fatigue (–), cortisol level (+)

(Continued to the next page)

Author (year)	Research design	Theoretical framework	Study participants: Pregnancy period (if primipara)/ High-risk factors	Study participants: Sample size	Intervention: Scope	Intervention: Setting /unit	Intervention: Name of intervention (contents)	Intervention: Methods (tools)	Intervention: Experimental period	Intervention: Sessions	Intervention: Time per session (minute)	Follow-up period	Outcome variables
Shin and Song [S38] (2005)	RCT	NA	During pregnancy (IUP 5-30 wks)/ Hyperemesis gravidarum	E1:23 E2:21 C:22	Women	Hospital /individual	Nei-guan (P6) acupressure for pregnant women with hyperemesis gravidarum	Acupressure	3–7 days	09–21	10	Hospital days #3,4, discharge date (1-6 days after intervention)	Nausea/ vomiting, (+), discomfort (+)
II-6. Aroma inhalation (n = 1)													
Go and Park [S39] (2017)	NRCT	NA	During pregnancy (IUP 20-34 wks)/ High-risk pregnancy	E:25 C:25	Women	Hospital /individual	Aroma inhalation therapy in high-risk pregnant women	Aroma inhalation	5 days	15	2	Immediately after intervention	Antenatal stress (+), state anxiety (–), depression (–), ANS (–)
III.	Psychosocial support (n = 6)												
III-1. Spouse support reinforcement (n = 3)													
Eom et al. [S40] (2012)	NRCT	NA	During pregnancy (IUP 37–42 wks)/None	E:62 C:60	Women and spouses	Hospital /individual	One-session spouse-support enhancement childbirth education (importance of spouse support on labor and delivery, physiology and psychology of labor, relaxation, breathing, imagery, focusing, empowerment)	Instruction, demonstration, practice and discussion (PPT, picture)	1 day	1	60	2 hours post-delivery	Perception of childbirth experience (+), self-efficacy (childbirth) (–)
Kwak et al. [S41] (2004)	NRCT	NA	During pregnancy (IUP 37–42 wks) (primipara) /None	E:81 C:92	Women and spouses	Hospital /individual	One-session spouse's support reinforcement prenatal education program (delivery process, spouse support method, relaxation, breathing and imagery)	Instruction and demonstration (booklet, picture, video)	1 day	1	60–120	1 day post-delivery	Spouse support (+), spouse participation (+)
Ahn [S42] (1985)	NRCT	NA	During pregnancy (IUP 28 wks) (primipara) /None	E:70 C:70	Women and spouses	Hospital /group	Husband's supportive behavior reinforcement education (spousal physical and psychological support behavior)	Instruction (animation, audio tape)	4 wks	6	25	4 wks post-intervention	Spouse support (+), antenatal stress (+)

(Continued to the next page)

KJWHN

Author (year)	Research design	Theoretical framework	Study participants: Pregnancy period (if primipara)/ High-risk factors	Study participants: Sample size	Intervention: Scope	Intervention: Setting /unit	Intervention: Name of intervention (contents)	Intervention: Methods (tools)	Intervention: Experimental period	Intervention: Sessions	Intervention: Time per session (minute)	Follow-up period	Outcome variables
III-2. Family participated delivery (n = 3)													
Oh et al. [S43] (2006)	NRCT	NA	During pregnancy & labor and birth (IUP 28–32 wks) (primipara) /None	E1:17 E2:20 C:20	Women and spouses or family	Hospital /individual +group	Childbirth education and family participated delivery in a labor-delivery-recovery room (Lamaze method, relaxation, breathing, imagery, normal delivery process, breastfeeding, newborn care, bathing, postpartum care)	Instruction and others	4 wks	4	120	1 hour post-delivery	State anxiety (–), labor pain (–), perception of childbirth experience (+)
Jang and Park [S44] (2002)	NRCT	NA	During labor and birth (IUP 37–42 wks) (primipara) /None	E:30 C:30	Women and spouses or family	Hospital /individual	Family-participated delivery in a labor delivery room	Others	1 day	1	-	> 3 hours post-delivery	Length of labor (+), labor pain (–), perception of childbirth experience (+)
Choi and Kim [S45] (2013)	NRCT	NA	During labor and birth (IUP 37–42 wks)/ Cesarean section	E:34 C:32	Spouse	Hospital /individual	Father participation program in cesarean section (explanation of procedure of cesarean section, handwashing, gowning, capping, asking, emotional support, infant attachment)	Instruction and others	1 day	1	37	3 days post-delivery	Father-infant attachment (+)

ANS: autonomic nervous system; BMI: body mass index; BP: blood pressure; BT: skin temperature; BW: body weight; C: control group; CD, compact disc; E: experimental group; FBS: fasting blood sugar; FHR: fetal heart rate; GDM: gestational diabetes mellitus; GROW: goal-reality-options-will; HbA1C: hemoglobin A1C; HC: head circumference; HR: heart rate; IMCHB: interaction model of client health behavior; IUP: intrauterine period; NA: not applicable; NRCT: non-randomized controlled trial; PPT: PowerPoint slides; PP2: postprandial 2-hour blood glucose test; RCT: randomized controlled trial; S^aO^2: oxygen saturation; wks: weeks.
